# Clinical Validation of Adjusted Corneal Power in Patients with Previous Myopic Lasik Surgery

**DOI:** 10.1155/2015/824293

**Published:** 2015-10-07

**Authors:** Vicente J. Camps, David P. Piñero, Veronica Mateo, Celia García, Alberto Artola, Rafael Pérez-Cambrodi, Pedro Ruiz-Fortes

**Affiliations:** ^1^Department of Optics, Pharmacology and Anatomy, University of Alicante, 03690 San Vicente del Raspeig, Spain; ^2^Foundation for the Visual Quality (FUNCAVIS), 03016 Alicante, Spain; ^3^Department of Ophthalmology, Oftalmar, Medimar International Hospital, 03016 Alicante, Spain

## Abstract

*Purpose*. To validate clinically a new method for estimating the corneal power (*P*_*c*_) using a variable keratometric index (*n*_*k*adj_) in eyes with previous laser refractive surgery. *Setting*. University of Alicante and Medimar International Hospital (Oftalmar), Alicante, (Spain). *Design*. Retrospective case series. *Methods*. This retrospective study comprised 62 eyes of 62 patients that had undergone myopic LASIK surgery. An algorithm for the calculation of *n*_*k*adj_ was used for the estimation of the adjusted keratometric corneal power (*P*_*k*adj_). This value was compared with the classical keratometric corneal power (*P*_*k*_), the True Net Power (TNP), and the Gaussian corneal power (*P*_*c*Gauss_). Likewise, *P*_*k*adj_ was compared with other previously described methods. *Results*. Differences between *P*_*c*Gauss_ and *P*_*c*_ values obtained with all methods evaluated were statistically significant (*p* < 0.01). Differences between *P*_*k*adj_ and *P*_*c*Gauss_ were in the limit of clinical significance (*p* < 0.01, loA [−0.33,0.60] D). Differences between *P*_*k*adj_ and TNP were not statistically and clinically significant (*p* = 0.319, loA [−0.50,0.44] D). Differences between *P*_*k*adj_ and previously described methods were statistically significant (*p* < 0.01), except with *P*_*c*HaigisL_ (*p* = 0.09, loA [−0.37,0.29] D). *Conclusion*. The use of the adjusted keratometric index (*n*_*k*adj_) is a valid method to estimate the central corneal power in corneas with previous myopic laser refractive surgery, providing results comparable to *P*_*c*HaigisL_.

## 1. Introduction

The precise measurement of corneal power after myopic laser refractive surgery is still currently an issue under debate. Several methods have been proposed during the last years which are classified as methods requiring historical clinical data and methods not requiring historical data. Among those methods requiring previous clinical data, some of them are based on a correction of the corneal power using the refracting change achieved [1–3] and others on performing such correction by adjusting the keratometric index [4–7]. The main disadvantage of all these methods is that they are infeasible if previous clinical patient's data are not available. For this reason, other methods that do not require patient's historical data have been developed [3, 5, 8–11]. In this line, our research group has recently proposed a new method for estimating with enough accuracy the corneal power using the keratometric approach that has been found to be valid in both healthy [12] and post-LASIK eyes [13]. This algorithm based on a variable keratometric index was named adjusted keratometric index (*n*_*k*adj_) and it has been prevalidated clinically in a sample of 32 eyes that had undergone previously myopic LASIK surgery [13]. The aim of the current study is to validate clinically this algorithm for the estimation of the corneal power in eyes with previous myopic LASIK in a larger population including also a larger range of intended refractive corrections.

## 2. Methods

### 2.1. Patients and Examination

This retrospective study comprised 62 eyes of 62 patients that had undergone previous correction of a myopic refractive error by means of laser in situ keratomileusis (LASIK) surgery. All LASIK surgeries had been performed using the Pulzar Z1 solid-state laser (CustomVis Laser Pty Ltd., Osborne Park, Australia, currently CV Laser Pty Ltd.) at the Department of Ophthalmology (Oftalmar) of the Vithas Medimar International Hospital (Alicante, Spain). All surgeries had been performed by one experienced surgeon (AA) between October 2012 and December 2013. For this study, only one eye from each subject was chosen according to a random number sequence (dichotomic sequence, 0 and 1). A comprehensive ophthalmologic examination was performed in all cases at least 3 months after surgery, which included refraction, corrected distance visual acuity (CDVA), slit lamp biomicroscopy, Goldman tonometry, fundus evaluation, and the analysis of the corneal structure by means of a Scheimpflug photography-based tomographer, the Pentacam system (Oculus Optikgeräte GmbH, Germany, software version 1.14r01). All patients were informed after surgery about this retrospective study and signed an informed consent document in accordance with the Helsinki Declaration.

### 2.2. Corneal Power Calculation

Our research group recently proposed the use of a variable keratometric index (*n*_*k*adj_) for the estimation of the corneal power (*P*_*c*_) using the keratometric approach in patients with previous myopic LASIK surgery [13]. The following expression was defined for *n*_*k*adj_ considering the ocular conditions of the Gullstrand eye model and the range of anterior and posterior curvature that is commonly found in this kind of patients [13]:

(1) $$\begin{array}{c} n_{kadj}=-0.0064286r_{1c}+1.37688, \end{array}$$

where *r*_1*c*_ is the postoperative anterior corneal radius.

Furthermore, adjusted keratometric corneal power (*P*_*k*adj_) was defined as follows [13]:

(2) $$\begin{array}{c} P_{kadj}=\frac{n_{kadj}-1}{r_{1c}}. \end{array}$$

For comparison purposes, the keratometric corneal power was also calculated using the classical keratometric index *n*_*k*_ = 1.3375 (*P*_*k*(1.3375)_).

The Gaussian corneal power was calculated using the following expression:

(3) PcGaussP1c+P2c−δP1cP2c=nc−nar1c+nha−ncr2c−ecnc·nc−nar1c·nha−ncr2c,

where *P*_*c*Gauss_ is the Gaussian total corneal power, *P*_1*c*_ is the anterior corneal power, *P*_2*c*_ is the posterior corneal power, *r*_1*c*_ is the anterior corneal radius, *r*_2*c*_ is the posterior corneal radius, *n*_*a*_ is the refractive index of air, *n*_*c*_ is the refractive index of the cornea, *n*_ha_ is the refractive index of the aqueous humour, and *e*_*c*_ is the central corneal thickness.

Likewise, the True Net Power (TNP) was also recorded, which is the corneal power provided by the Pentacam system (Oculus) based on the anterior (*r*_1*c*_) and posterior (*r*_2*c*_) corneal radius and calculated by using the Gaussian equation (*P*_*c*Gauss_) with the Gullstrand eye model, but neglecting the corneal thickness (*e*_*c*_):

(4) True  net  Power  TNP=1.376−1r1c·1000+1.336−1.376r2c·1000.

Besides this, corneal power was also estimated by using other methods described previously for such purpose in eyes with previous myopic laser refractive surgery:

(1) Methods requiring previous clinical data:
  (a) Awwad method [3]:
    (5) $$\begin{array}{c} {\text{P}}_{cAwwad}=P_{c}-0.23\Delta SE; \end{array}$$
  (b) Camellin method [4]:
    (6) $$\begin{array}{c} P_{cCamellin}=\frac{\left[\left(1.3319+0.00113\Delta SE\right)-1\right]}{\left(r_{1cpost}/1000\right)}; \end{array}$$
  (c) Clinical History method:
    (7) $$\begin{array}{c} P_{cpost}=P_{cpre}+\Delta SE; \end{array}$$
  (d) Jarade method [4]:
    (8) $$\begin{array}{c} P_{cJarade}=\frac{\left[\left(1.3375+0.0014\Delta SE\right)-1\right]}{\left(r_{1cpost}/1000\right)}; \end{array}$$
  (e) Savini method [5, 6]:
    (9) $$\begin{array}{c} P_{cSavini}=\frac{\left[\left(1.338+0.0009856\Delta SE\right)-1\right]}{\left(r_{1cpost}/1000\right)}. \end{array}$$
(2) Methods not requiring previous data:
  (a) Haigis-L method:
    (10) $$\begin{array}{c} P_{cHaigisL}=-5.1625r_{1cpost}+82.2603-0.35; \end{array}$$
  (b) Shammas method [8]:
    (11) $$\begin{array}{c} P_{cShammas}=1.14\left(\;P_{c}-6.8\;\right); \end{array}$$
  (c) Seitz method [6]:
    (12) $$\begin{array}{c} P_{cSeitz}=1.114\left(\;P_{c}-4.98\;\right), \end{array}$$

where ΔSE = SE_pre_ − SE_post_, SE_pre_ and SE_post_ being the pre- and postsurgery spherical equivalents, *r*_1*c*post_ is the postsurgery anterior corneal radius, and *P*_*c*pre_ is the presurgery keratometric corneal power.

For the clinical validation of *P*_*k*adj_, it was compared with *P*_*c*Gauss_ and TNP. Likewise, the different methods mentioned above were also compared with *P*_*k*adj_ and *P*_*c*Gauss_ in order to demonstrate which was the most accurate approach.

### 2.3. Statistical Analysis

Statistical analysis was performed using the software SPSS version 19.0 for Windows (SPSS, Chicago, Illinois, USA). Normality of all data distributions was first confirmed by means of the Kolmogorov-Smirnov test. Specifically, the paired Student *t*-test or Wilcoxon test was used for comparing the different methods of *P*_*c*_ calculation depending on whether the normality condition could be assumed or not. The Bland-Altman analysis [14] was used for evaluating the agreement and interchangeability of the different methods for obtaining the corneal power.

## 3. Results

This study comprised 62 eyes of 62 patients (34 women [54.8%]), with a mean age of 33.42 ± 7.16 years (range 21 to 52 years) and with preoperative myopia between −0.25 and −6.8 D. The sample comprised 31 left eyes (50%). Mean ocular features of the eyes evaluated in the current study can be seen in Table 1.  Table 2 shows the values of corneal power estimated with the previously published methods (Awwad, Camellin, Clinical History, Haigis-L, Jarade, Savini, Seitz, and Shammas methods).

### 3.1. Clinical Validation of *P*_*k*adj_

As shown in Table 3, there were significant differences (*p* < 0.01, paired Student's *t*-test) between *P*_*k*adj_ and *P*_*c*Gauss_, but not (*p* = 0.319, paired Student's *t*-test) between *P*_*k*adj_ and TNP. The Bland-Altman analysis showed that differences between *P*_*k*adj_ and *P*_*c*Gauss_ were barely clinically significant (mean difference: 0.14 ± 0.24; limits of agreement (loA): [−0.33, 0.60 D]) and that differences between *P*_*k*adj_ and TNP were not clinically significant (mean difference 0.03 ± 0.24; loA: [−0.50,0.44 D]). A very strong and statistically significant correlation was found between *P*_*k*adj_ and *P*_*c*Gauss_ (*r* = 0.994, *p* < 0.01) as well as between *P*_*k*adj_ and TNP (*r* = 0.994, *p* < 0.01).

### 3.2. Comparison between *P*_*k*adj_ and Corneal Power Values Estimated with Other Methods

As shown also in Table 3, differences between *P*_*k*adj_ and the rest of the methods for estimation of *P*_*c*_ were statistically significant (*p* < 0.01), except for the difference between *P*_*k*adj_ and *P*_*c*HaigisL_ (*p* = 0.09). The Bland-Altman analysis confirmed that all these statistically significant differences were also clinically relevant as the ranges of agreement were quite large (>±0.5 D). Only differences between *P*_*k*adj_ and *P*_*c*HaigisL_ (mean: −0.04 ± 0.17 D) did not reach clinical significance (loA: [−0.37 to 0.29 D]).

Table 4 summarizes the results of the comparison between the corneal power calculated considering the curvature of the two corneal surfaces as well as corneal thickness (*P*_*c*Gauss_) and those values obtained with the other previously published methods for corneal power estimation in corneas with previous myopic laser refractive surgery. As shown, all differences between *P*_*c*Gauss_ and *P*_*c*_ values obtained with such methods were statistically significant (*p* < 0.01). Considering the Bland-Altman analysis, *P*_*k*adj_ and *P*_*c*HaigisL_ provided the lower mean differences with *P*_*c*Gauss_ (−0.14 ± 0.24 D and −0.18 ± 0.21 D, resp.) and the smaller ranges of agreement ([−0.6 to 0.33] and [−0.60 to 0.23], resp.). *P*_*c*Camellin_ was also close to *P*_*c*Gauss_ (mean difference: 0.42 ± 0.14 D), but the range of agreement was larger than that found for *P*_*k*adj_ and *P*_*c*HaigisL_ ([0.15 to 0.68 D]).

## 4. Discussion

In the current study, the use of *P*_*k*adj_ as a method to estimate the corneal power in corneas with previous myopic laser refractive surgery has been validated clinically. Furthermore, the results show that previously reported methods for this estimation are not better than the calculation of *P*_*k*adj_. We have confirmed the clinical prevalidation we conducted in 32 eyes that had undergone myopic LASIK surgery where no statistically significant differences between *P*_*k*adj_ and TNP were found, with a mean difference value of 0.00 D and limits of agreement of −0.45 and +0.46 D [13].

In the current series, a mean difference of 0.03 ± 0.24 D between TNP and *P*_*k*adj_ (*p* = 0.319) and a range of agreement from −0.50 to 0.44 D have been found. Besides this, the comparison of *P*_*k*adj_ with *P*_*c*Gauss_ has been also performed. It should be considered that *P*_*c*Gauss_ takes into account neither the curvature of the two corneal surfaces nor the corneal thickness. In this comparison, the mean difference between *P*_*k*adj_ and *P*_*c*Gauss_ was 0.14 ± 0.24 D (*p* < 0.01), with a range of agreement from −0.33 to 0.60 D. There were only 4 cases out of 66 that showed differences between *P*_*k*adj_ and *P*_*c*Gauss_ of more than ±0.5 D. All these results are consistent with the theoretical predictions reported previously that estimated maximum differences between *P*_*k*adj_ and *P*_*c*Gauss_ of ±0.7 D [13]. Therefore, *P*_*k*adj_ is an acceptable method for estimating the corneal power of corneas with previous myopic laser refractive surgery as 100% of estimations were within ±0.7 D if *P*_*c*Gauss_ is taken as reference. When *P*_*k*adj_ was compared with other different methods of corneal power estimation, statistically significant differences were found (*p* < 0.01), except for the comparison between *P*_*k*adj_ and *P*_*c*HaigisL_ (*p* = 0.09). The comparison between *P*_*c*HaigisL_ and *P*_*k*adj_ showed a mean of difference of −0.04 ± 0.17 and a range of agreement from −0.37 to 0.29 D, confirming that *P*_*k*adj_ and *P*_*c*HaigisL_ were interchangeable.

Considering that *P*_*c*Gauss_ is the most exact method of calculation of the central corneal power in paraxial optics and that *P*_*k*adj_, *P*_*c*HaigisL_, and *P*_*c*Gauss_ can be considered interchangeable according to the results of our study, *P*_*k*adj_ and *P*_*c*HaigisL_ can be considered appropriate methods for estimating the corneal power in corneas with previous myopic laser refractive surgery when posterior corneal surface data are unknown in clinical practice. The remaining methods (independently if previous historical data are required or not) were found to overestimate *P*_*c*Gauss_ (Table 4). The classical keratometric approach for corneal power estimation based on calculations performed only considering the anterior corneal radius (*P*_*k*(1.3375)_) induces significant overestimations, ranging from 1.07 to 1.97 D. However, this was not the method providing the poorest performance. The Clinical History method was the most variable procedure, providing corneal power estimations that differed from *P*_*c*Gauss_ in a range going from −1.55 to 3.76 D. Among methods requiring previous historical clinical data, *P*_*c*Camellin_ was the method providing lower overestimations of corneal power, with differences compared to *P*_*c*Gauss_ ranging from 0.04 to 0.78 D. Furthermore, this corneal power estimation method could be clinically valid by using a correction factor. The rest of the methods evaluated led to overestimations of more than 1 D.

Among methods requiring previous historical clinical data, *P*_*c*Camellin_, *P*_*c*Savini_, and *P*_*c*Jarade_ methods are based on a variation of the keratometric refractive index depending on the induced refractive change (ΔSE). This keratometric approach for corneal power estimation is more appropriate than the use of a single value of keratometric index in all cases, but it is still associated with some level of limitation, as has been shown in the current study. A specific keratometric index value for each cornea must be calculated if the keratometric approach is intended to be used for an estimation of corneal power, as demonstrated by our research group in a previous study [13]. This keratometric index was named as exact keratometric index (*n*_*k*exact_) and its calculation requires the measurement of anterior and posterior corneal curvature [13]. As devices measuring the posterior corneal curvature are not available in all clinical settings, our group developed the concept of adjusted keratometric index (*n*_*k*adj_), which is a clinically valid method for estimating an appropriate keratometric index allowing calculation of the corneal power with enough accuracy. This method only requires the measurement of the anterior corneal radius postoperatively, which can be easily obtained in any clinical setting.

Among methods not requiring previous historical clinical data, the methods evaluated in the current study led to overestimations of more than 1 D except for *P*_*c*HaigisL_, as commented previously. These findings confirm the relevance of the curvature of the posterior corneal surface in the error associated with the corneal power estimation when the keratometric approach is used. This is consistent with previous scientific evidence remarking that there is an error in considering the *k* = *r*_2*c*_/*r*_1*c*_ ratio as a constant when corneal power is estimated in eyes after refractive surgery using the keratometric approach [15]. This is also the reason explaining the inaccuracy of methods of corneal power estimation based on considering the refractive change induced. Our results also confirm that the use of an inappropriate method for corneal power estimation in eyes with previous laser refractive surgery mainly leads to an overestimation of corneal power and consequently to an underestimation of the IOL power required when cataract surgery is planned in this type of cases, resulting in hyperopic residual refractive errors postoperatively.

In conclusion, the use of the adjusted keratometric index (*n*_*k*adj_) is a valid and easy method to estimate the central corneal power in corneas with previous myopic laser refractive surgery, improving the accuracy of methods described previously for such purpose. Only *P*_*c*HaigisL_ has been found to be comparable to our method and therefore leading to differences compared to the Gaussian corneal power which are clinically acceptable. The advantage of the use of *P*_*k*adj_ method is that the estimation of corneal power can be performed with the only requirement of measuring the postoperative anterior corneal radius and with an associated error within ±0.7 D and only 6% of cases showing differences with the Gaussian power out of the range of ±0.5 D.

## 5. Conclusion

With this paper, a new method for calculating corneal power after myopic refractive surgery was validated clinically, with the advantage that only postoperative refractive surgery parameters are required.

## Figures and Tables

**Table 1:** Mean ocular features of the eyes evaluated in the current study.

Parameter	Mean ± SD	Range
SE_pre_ (D)	−3.0 ± 1.6	−6.8 to 0.0
SE_post_ (D)	0.1 ± 0.3	0.0 to 1.0
*r*_1*c*pre_ (mm)	7.70 ± 0.25	7.2 to 8.3
*r*_1*c*post_ (mm)	8.18 ± 0.34	7.5 to 9.3
*r*_2*c*pre_ (mm)	6.34 ± 0.24	5.9 to 6.9
*r*_2*c*post_ (mm)	6.37 ± 0.24	5.8 to 7.0
*e*_*c*_ (*μ*m)	512 ± 37	407 to 590

SE_pre_ = preoperative spherical equivalent; SE_post_ = postoperative spherical equivalent; *r*_1*c*pre_ = preoperative radius of curvature of the anterior corneal surface; *r*_1*c*post_ = postoperative radius of curvature of the anterior corneal surface; *r*_2*c*pre_ = preoperative radius of curvature of the posterior corneal surface; *r*_2*c*post_ = postoperative radius of curvature of the posterior corneal surface; *e*_*c*_ = corneal thickness.

**Table 2:** Corneal power measured with different methods.

*P*_*c*_ calculation method	Mean ± SD	Range
*P*_*k*adj_ (D)	39.70 ± 1.86	34.21 to 43.59
*P*_*c*Gauss_ (D)	39.84 ± 1.72	34.58 to 43.38
TNP (D)	39.73 ± 1.71	34.49 to 43.26
*P*_*k*(1.3375)_ (D)	41.31 ± 1.66	36.34 to 44.79

*Methods requiring previous data*		
*P*_*c*Awwad_ (D)	40.60 ± 1.92	34.84 to 44.53
*P*_*c*Cammellin_ (D)	40.25 ± 1.74	35.07 to 43.90
*P*_*c*CHM_ (D)	40.79 ± 2.11	33.94 to 44.80
*P*_*c*Jarade_ (D)	40.81 ± 1.80	35.45 to 44.58
*P*_*c*Savini_ (D)	41.00 ± 1.77	35.72 to 44.71

*Methods not requiring previous data*		
*P*_*c*HaigisL_ (D)	39.66 ± 1.74	34.03 to 43.01
*P*_*c*Seitz_ (D)	41.04 ± 1.85	35.56 to 44.92
*P*_*c*Shammas_ (D)	40.29 ± 1.90	34.68 to 44.26

*P*_*k*adj_ = adjusted keratometric corneal power; *P*_*c*Gauss_ = Gaussian corneal power; TNP = True Net Power; *P*_*k*(1.3375)_ = keratometric corneal power using *n*_*k*_ = 1.3375; *P*_*c*Awwad_ = corneal power obtained using Awwad formula; *P*_*c*Cammellin_ = corneal power obtained using Camellin formula; *P*_*c*CHM_ = corneal power obtained using Clinical History method; *P*_*c*HaigisL_ = corneal power obtained using Haigis-L formula; *P*_*c*Jarade_ = corneal power obtained using Jarade formula; *P*_*c*Savini_ = corneal power obtained using Savini formula;  *P*_*c*Seitz_ = corneal power obtained using Seitz formula; *P*_*c*Shammas_ = corneal power obtained using Shammas formula.

**Table 3:** Bland and Altman analysis outcomes of the comparison between *P*_*k*adj_ and the corneal power obtained with other methods.

	Δ*P*_*c*_ ± SD (D)	LoA (D)	Range (D)	*p* value
*P*_*c*Gauss_ – *P*_*k*adj_ (D)	0.14 ± 0.24	−0.33 to 0.60	−0.43 to 0.70	<0.01
TNP – *P*_*k*adj_ (D)	0.03 ± 0.24	−0.50 to 0.44	−0.55 to 0.61	0.319
*P*_*k*(1.3375)_ – *P*_*k*adj_ (D)	1.61 ± 0.19	1.23 to 1.99	1.20 to 2.18	<0.01

*Methods requiring previous data*				
*P*_*c*Awwad_ – *P*_*k*adj_ (D)	0.90 ± 0.32	0.26 to 1.53	0.04 to 1.65	<0.01
*P*_*c*Camellin_ – *P*_*k*adj_ (D)	0.56 ± 0.20	0.15 to 0.96	0.04 to 1.02	<0.01
*P*_*c*CHM_ – *P*_*k*adj_ (D)	1.09 ± 0.84	−0.55 to 2.73	−1.13 to 3.96	<0.01
*P*_*c*Jarade_ – *P*_*k*adj_ (D)	1.11 ± 0.23	0.67 to 1.55	0.49 to 1.65	<0.01
*P*_*c*Savini_ – *P*_*k*adj_ (D)	1.30 ± 0.19	0.93 to 1.67	0.80 to 1.72	<0.01

*Methods not requiring previous data*				
*P*_*c*HaigisL_ – *P*_*k*adj_ (D)	−0.04 ± 0.17	−0.37 to 0.29	−0.58 to 0.12	0.09
*P*_*c*Seitz_ – *P*_*k*adj_ (D)	1.34 ± 0.00	1.33 to 1.35	1.32 to 1.35	<0.01
*P*_*c*Shammas_ – *P*_*k*adj_ (D)	0.59 ± 0.04	0.52 to 0.67	0.48 to 0.67	<0.01

*P*_*k*adj_ = adjusted keratometric corneal power; *P*_*c*Gauss_ = Gaussian corneal power; TNP = True Net Power; *P*_*k*(1.3375)_ = keratometric corneal power using *n*_*k*_ = 1.3375; *P*_*c*Awwad_ = corneal power obtained using Awwad formula; *P*_*c*Cammellin_ = corneal power obtained using Camellin formula; *P*_*c*CHM_ = corneal power obtained using Clinical History method; *P*_*c*HaigisL_ = corneal power obtained using Haigis-L formula; *P*_*c*Jarade_ = corneal power obtained using Jarade formula; *P*_*c*Savini_ = corneal power obtained using Savini formula; *P*_*c*Seitz_ = corneal power obtained using Seitz formula; *P*_*c*Shammas_ = corneal power obtained using Shammas formula.

**Table 4:** Bland and Altman analysis outcomes of the comparison between *P*_*c*Gauss_ and the corneal power obtained with other methods.

	Δ*P*_*c*_ ± SD (D)	LoA (D)	Range (D)	*p* value
*P*_*k*adj_ – *P*_*c*Gauss_ (D)	−0.14 ± 0.24	−0.60 to 0.33	−0.70 to 0.43	<0.01
TNP – *P*_*c*Gauss_ (D)	−0.11 ± 0.01	−0.13 to −0.09	−0.08 to −0.13	<0.01
*P*_*k*(1.3375)_ – *P*_*c*Gauss_ (D)	1.47 ± 0.19	1.10 to 1.84	1.07 to 1.97	<0.01

*Methods requiring previous data*				
*P*_*c*Awwad_ – *P*_*c*Gauss_ (D)	0.76 ± 0.29	0.18 to 1.34	−0.28 to 1.26	<0.01
*P*_*c*Camellin_ – *P*_*c*Gauss_ (D)	0.42 ± 0.14	0.15 to 0.68	0.04 to 0.78	<0.01
*P*_*c*CHM_ – *P*_*c*Gauss_ (D)	0.95 ± 0.83	−0.67 to 2.58	−1.55 to 3.76	<0.01
*P*_*c*Jarade_ – *P*_*c*Gauss_ (D)	0.97 ± 0.18	0.63 to 1.32	0.40 to 1.36	<0.01
*P*_*c*Savini_ – *P*_*c*Gauss_ (D)	1.16 ± 0.14	0.88 to 1.45	0.70 to 1.49	<0.01

*Methods not requiring previous data*				
*P*_*c*HaigisL_ – *P*_*c*Gauss_ (D)	−0.18 ± 0.21	−0.60 to 0.23	−0.71 to 0.40	<0.01
*P*_*c*Shammas_ – *P*_*c*Gauss_ (D)	0.45 ± 0.26	−0.06 to 0.97	−0.18 to 1.04	<0.01
*P*_*c*Seitz_ – *P*_*c*Gauss_ (D)	1.20 ± 0.23	0.74 to 1.66	0.64 to 1.67	<0.01

*P*_*k*adj_ = adjusted keratometric corneal power; *P*_*c*Gauss_ = Gaussian corneal power; TNP = True Net Power; *P*_*k*(1.3375)_ = keratometric corneal power using *n*_*k*_ = 1.3375; *P*_*c*Awwad_ = corneal power obtained using Awwad formula; *P*_*c*Cammellin_ = corneal power obtained using Camellin formula; *P*_*c*CHM_ = corneal power obtained using Clinical History method; *P*_*c*HaigisL_ = corneal power obtained using Haigis-L formula; *P*_*c*Jarade_ = corneal power obtained using Jarade formula; *P*_*c*Savini_ = corneal power obtained using Savini formula; *P*_*c*Seitz_ = corneal power obtained using Seitz formula; *P*_*c*Shammas_ = corneal power obtained using Shammas formula.
